# Epidemiological trends in HIV infection among female sex workers in Cabo Verde, 2013-2023: evidence from three integrated bio-behavioural surveys

**DOI:** 10.11604/pamj.2026.53.172.50672

**Published:** 2026-04-24

**Authors:** Adilson José DePina, Nelson Neide Furtado Ribeiro, Maria Celina Moreira Ferreira, Marta Freire, José Manuel Marques, Artur Jorge Correia

**Affiliations:** 1 *Comitê de Coordenação do Combate à SIDA*, Ministry of Health, Praia, Cabo Verde,; 2 National Program to Combat HIV, Ministry of Health, Praia, Cabo Verde,; 3 Analyses Ltd, Business Consulting, Praia, Cabo Verde

**Keywords:** HIV prevalence, female sex workers, integrated bio-behavioural survey, stigma, discrimination, Cabo Verde

## Abstract

**Introduction:**

female sex workers (FSWs) remain disproportionately affected by human immunodeficiency virus (HIV) infection globally. In Cabo Verde, where the population HIV prevalence is below 1%, targeted surveillance among key populations is essential to sustain epidemic control. This study aimed to assess temporal trends in HIV infection prevalence, age-specific and geographic patterns, and behavioural and structural determinants of HIV infection among female sex workers in Cabo Verde between 2013 and 2023.

**Methods:**

three cross-sectional integrated bio-behavioural surveys were in 2013 (n=452), 2017 (n=481), and 2023 (n=519) using respondent-driven sampling in six municipalities. Data were collected using structured interviewer-administered questionnaires and rapid HIV testing. Descriptive statistics, correlation analyses, and multivariable logistic regression were used to identify factors associated with HIV infection.

**Results:**

HIV prevalence remained stable at 5.4% (2013), 5.3% (2017), and 4.9% (2023), far exceeding national levels (∼0.6%). Although HIV infection prevalence declined slightly over time (5.4% to 4.9%), this trend was not statistically significant (p for trend = 0.21). HIV infection prevalence was higher among older FSWs (30-44 years; 14.7%) and those with no formal education (23.1%). Age correlated positively with HIV (ρ=0.22, p<0.01), while education and condom use were inversely correlated. Older age (aOR=3.7, 95% CI: 1.9-6.9) and reported stigma (aOR=1.8, 95% CI: 1.1-3.0) independently predicted infection.

**Conclusion:**

HIV prevalence among FSWs in Cabo Verde has remained unchanged for a decade despite improved testing coverage (96% in 2023). Persistent structural inequities and stigma underscore the need for rights-based interventions, expanded PrEP, and strengthened prevention services for key populations.

## Introduction

Female sex workers (FSWs) remain among the populations most disproportionately affected by the global HIV epidemic. Epidemiological evidence consistently demonstrates that HIV prevalence among FSWs can be up to 30 times higher than that of the general population, reflecting the intersection of biological risk, gender inequality, stigma, and persistent structural vulnerabilities that limit access to prevention and care services [1,2]. Despite substantial global advances in HIV prevention and treatment, including the scale-up of antiretroviral therapy (ART) and the introduction of new HIV prevention protocols such as pre-exposure prophylaxis (PrEP), FSWs continue to experience markedly higher HIV incidence and prevalence compared with other population groups [3,4].

In 2022, an estimated 39 million people were living with HIV infection globally, of whom approximately 29.8 million were receiving ART [5]. The expansion of ART has contributed to a greater than 55% decline in AIDS-related mortality over the past two decades; however, progress remains uneven, particularly among key populations [5,6]. Persistent gaps in HIV prevention, testing, and treatment coverage among FSWs, men who have sex with men (MSM), transgender women, and people who inject drugs (PWID) continue to undermine efforts to achieve global targets to end AIDS as a public health threat by 2030 [7].

Sub-Saharan Africa bears the largest share of the global HIV burden, accounting for more than 60% of all people living with HIV worldwide [5,8]. Within this region, HIV prevalence among FSWs frequently exceeds 20% in several national contexts, including Côte d´Ivoire, Nigeria, and Uganda [9-10]. These elevated rates are driven not only by biological susceptibility but also by deeply entrenched structural factors such as the criminalization of sex work, exposure to gender-based violence, economic insecurity, high levels of mobility, and limited access to HIV prevention methods such as condoms and PrEP, and non-discriminatory health services [11-13]. Although many African countries have achieved declines in population-level HIV incidence in recent years, gains among FSWs and other key populations have been comparatively modest, perpetuating ongoing transmission dynamics that threaten broader epidemic control [14].

Comparative experiences from other regions further highlight the importance of targeted and context-specific interventions. In South and Southeast Asia, focused HIV prevention strategies among FSWs, such as peer-led condom promotion, community empowerment models, and drop-in centers, have contributed to measurable reductions in HIV prevalence, although coverage and sustainability remain heterogeneous [15,16]. In the Caribbean and other small island developing states (SIDS), HIV epidemics remain highly concentrated among FSWs despite relatively low general population prevalence, reflecting challenges related to geographic dispersion, fragile outreach systems, and heightened social marginalization [17,18]. These settings underscore the distinct vulnerabilities faced by FSWs in small, resource-constrained contexts where sustaining specialized services for key populations competes with broader health system priorities.

Addressing HIV among FSWs, therefore, requires a comprehensive approach that integrates biomedical, behavioral, and structural interventions. The World Health Organization recommends combination prevention packages for FSWs that include consistent condom promotion, PrEP, harm reduction where applicable, regular voluntary HIV testing, linkage to ART, and interventions to reduce stigma and discrimination [19]. However, implementation of these recommendations remains inconsistent, constrained by limited political commitment, inadequate financing, and poor integration of services. Evidence from West Africa and the Caribbean indicates that without parallel structural reforms, such as decriminalization of sex work, violence prevention, and universal access to equitable healthcare, biomedical interventions alone are insufficient to reverse persistent disparities in HIV outcomes among FSWs [13,17,20].

In this context, robust and context-specific epidemiological data are essential to inform effective HIV prevention and control strategies specifically tailored to the needs of FSWs. Longitudinal surveillance through integrated bio-behavioural surveys (IBBS) provides a critical platform for monitoring trends in HIV prevalence, risky behaviours, and service uptake, while also elucidating the structural barriers that shape vulnerability and access to care. Analysis of IBBS data is therefore vital not only for guiding national policy and programmatic decisions, but also for advancing the global commitment to equity and to leaving no one behind in the pursuit of HIV elimination. The objectives of this study were to: (1) assess temporal trends in HIV prevalence among FSWs in Cabo Verde between 2013 and 2023; (2) examine age-specific and geographic patterns of HIV prevalence; and (3) identify behavioural and structural factors associated with HIV infection. Specifically, this study sought to answer the following research questions: (i) has HIV prevalence among FSWs changed over the past decade? (ii) which age groups and municipalities experience the highest HIV burden? and (iii) what individual and structural determinants are independently associated with HIV infection among FSWs?

## Methods

**Study design and setting:** the same standardized IBBS protocol was used across all three survey rounds, in 2013, 2017, and 2023, to ensure methodological comparability over time. The survey employed a cross-sectional design using standardized surveillance protocols adapted from World Health Organization (WHO) and Joint United Nations Programme on HIV/AIDS (UNAIDS) guidelines for HIV surveillance among key populations [21,22]. The surveys were designed to generate comparable estimates of HIV prevalence and associated risk factors over time. Data collection was conducted in six urban municipalities (Praia, Santa Catarina, Santa Cruz, São Vicente, Sal, and Boa Vista) selected based on prior mapping exercises, population mobility patterns, and documented concentrations of sex work activity across the archipelago. These municipalities collectively represent the primary urban and tourism hubs in Cabo Verde and account for the majority of reported sex work venues and client flows.

**Study population and eligibility criteria:** participants were eligible if they self-identified as female sex workers, were aged 15 years or older, reported having exchanged sex for money or goods within the six months preceding the survey, and resided or worked in one of the selected municipalities at the time of data collection. Women were not included if they did not self-identify as FSWs, had not exchanged sex for money or goods in the past six months, or were unable to provide informed consent.

**Sampling strategy and sample size:** personal network size was measured by asking participants the number of other FSWs they knew and had contact with within the past month. Respondent-driven sampling (RDS) weights were applied to prevalence estimates to account for differential network sizes. Equilibrium was assessed for key variables (age group and municipality) and was achieved within 4-6 recruitment waves in all survey rounds. Design effects were assumed in sample size calculations, and robust standard errors were used in regression analyses. Each seed received three recruitment coupons, consistent with standard RDS methodology to balance recruitment depth and network saturation [23]. Recruitment proceeded through multiple waves until equilibrium was achieved and target sample sizes were reached. Sample size calculations were based on HIV prevalence estimates from prior IBBS rounds, assuming a 95% confidence level, a design effect of 2.0, and an expected HIV prevalence ranging from 5% to 10%. These assumptions yielded target sample sizes of approximately 400-500 participants per survey round.

**Data collection procedures:** data were collected using a standardized IBBS questionnaire adapted from the WHO/UNAIDS key population surveillance instruments that were harmonized across the three survey rounds to allow valid temporal comparisons. Interviews were conducted in private and secure locations to ensure confidentiality and to encourage accurate reporting of sensitive information.

**Study variables:** the questionnaires captured detailed information on participants´ sociodemographic characteristics, including age, marital status, education level, employment status, and number of dependents, as well as sexual behaviours such as age at entry into sex work, number and types of clients and partners, and condom use with both paying and non-paying partners. Information was also collected on alcohol and illicit drug use, knowledge and attitudes related to HIV and other sexually transmitted infections, and access to and utilization of HIV prevention and treatment services, including HIV testing history, STI screening and treatment, and access to prevention commodities. In addition, participants were asked about experiences of stigma and discrimination, including family rejection and encounters with discrimination within healthcare settings. All interviews were conducted by trained female interviewers with experience working with key populations. Interviewers received standardized training on survey procedures, ethical conduct, and confidentiality. Field supervisors conducted regular monitoring and quality control checks throughout the data collection period to ensure consistency, completeness, and accuracy of the data collected.

**Laboratory procedures:** HIV testing was conducted using Determine™ HIV-1/2 as the screening test and Uni-Gold™ HIV as the confirmatory test, following the national HIV testing algorithm. The same testing algorithm was used across all three rounds. Reactive results were confirmed using a second rapid test, as per national guidelines. All participants received the same-day results along with pre- and post-test counseling. Individuals who tested HIV-positive were referred to designated HIV care and treatment services in line with Ministry of Health protocols [23].

**Statistical analysis:** data were double-entered, cleaned, and analyzed using Stata version 17.0 (StataCorp, College Station, TX, USA). Descriptive statistics were used to summarize sociodemographic characteristics and behavioral variables across survey rounds. HIV prevalence estimates and corresponding 95% confidence intervals (95% CI) were calculated for each survey year at the national level and stratified by municipality. Direct estimation of HIV incidence was not possible due to the cross-sectional design and absence of recency testing algorithms. As such, age-specific prevalence was used as a proxy indicator of cumulative exposure to HIV risk over time. Bivariate analyses were conducted using Pearson´s chi-square tests for categorical variables, and Student's t-tests were used for comparisons of continuous variables after confirming approximate normal distribution and adequate subgroup sample sizes (n > 30). Spearman´s rank correlation coefficients were used to assess associations between HIV status and cross-sectional surveys; predictors were not measured longitudinally in the same individuals; therefore, associations represent population-level relationships rather than causal trajectories.

Multivariable logistic regression models were fitted to identify independent predictors of HIV infection. Adjusted odds ratios (aORs) with 95% CIs were reported, controlling for potential confounders including age, education, condom use, alcohol and drug use, and municipality of residence or work. Temporal trends in HIV prevalence across survey rounds were assessed using Cochran-Armitage trend tests. To explore structural determinants of service uptake, additional analyses examined the association between stigma indicators (family rejection and reported discrimination in healthcare settings) and HIV testing uptake using logistic regression models. Interaction terms were tested to assess potential effect modification by age group and education level. Statistical significance was set at p < 0.05.

**Ethical considerations:** all study protocols were reviewed and approved by the National Committee of Ethics in Health Research of Cabo Verde (*Comité Nacional de Ética em Pesquisa em Saúde, CNEPS*), with the approval of 31/October/2013, the Deliberation 59/206, and CNEPS/2023/014.

**Written informed consent**: it was obtained from all participants before enrollment. For participants aged under 18 years, a waiver of parental consent was granted by the ethics committee due to the sensitive nature of sex work and the need to protect participant confidentiality. For participants aged 15-17 years, trained counselors provided youth-appropriate counseling, and referral pathways to adolescent-friendly sexual and reproductive health services were established. Participation was voluntary, and no parental consent was required, as approved by the ethics committee. Participation was voluntary, and no personal identifiers were collected. Recruitment through RDS coupons ensured anonymized referral chains. All participants received HIV counseling, and referrals were provided for HIV care, STI treatment, and psychosocial support services as appropriate.

## Results

Table 1 presents the sociodemographic characteristics of female sex workers across the three survey rounds. The age distribution shifted gradually toward older groups, with the proportion of women aged 35-44 years increasing from 20.2% in 2013 to 23.4% in 2017 and 28.6% in 2023, while those aged 15-24 years declined from 41.1% in 2013 to 37.2% in 2017 and 32.3% in 2023. Educational attainment remained low, with women reporting no formal education increasing slightly from 18.5% in 2013 to 21.7% in 2023, and only about 35% having secondary or higher education across all rounds. The geographic distribution was stable, with Praia consistently representing about 30-32% of participants, followed by São Vicente (~19%) and Sal (~16-18%). Regarding venue type, street-based sex work declined from 38.6% in 2013 to 34.2% in 2023, while bar/club-based and tourism-related venues increased from 51.5% in 2013 to 57.5% in 2023.

**Table 1:** geographic variation in condom use with the last paying client and HIV prevalence among female sex workers in Cabo Verde, 2023

Characteristic	2013 (n = 452)	2017 (n = 481)	2023 (n = 519)
**Age group (years)**			
15–19	14.8%	12.5%	10.2%
20–24	26.3%	24.7%	22.1%
25–29	21.5%	20.8%	19.4%
30–34	17.2%	18.6%	19.7%
35–39	12.4%	14.3%	16.8%
40–44	7.8%	9.1%	11.8%
**Education level**			
No formal education	18.5%	20.2%	21.7%
Primary	46.2%	44.6%	43.1%
Secondary or higher	35.3%	35.2%	35.2%
**Municipality**			
Praia	32.4%	31.1%	30.6%
São Vicente	18.7%	19.3%	18.9%
Sal	15.6%	16.8%	17.5%
Boa Vista	12.1%	12.7%	13.4%
Santa Cruz	11.3%	10.5%	10.2%
Santa Catarina	9.9%	9.6%	9.4%
**Venue type**			
Street-based	38.6%	36.9%	34.2%
Bar/club	34.1%	35.7%	36.8%
Hotel/tourism venue	17.4%	18.9%	20.7%
Other (home/online)	9.9%	8.5%	8.3%

**Figure 1:**
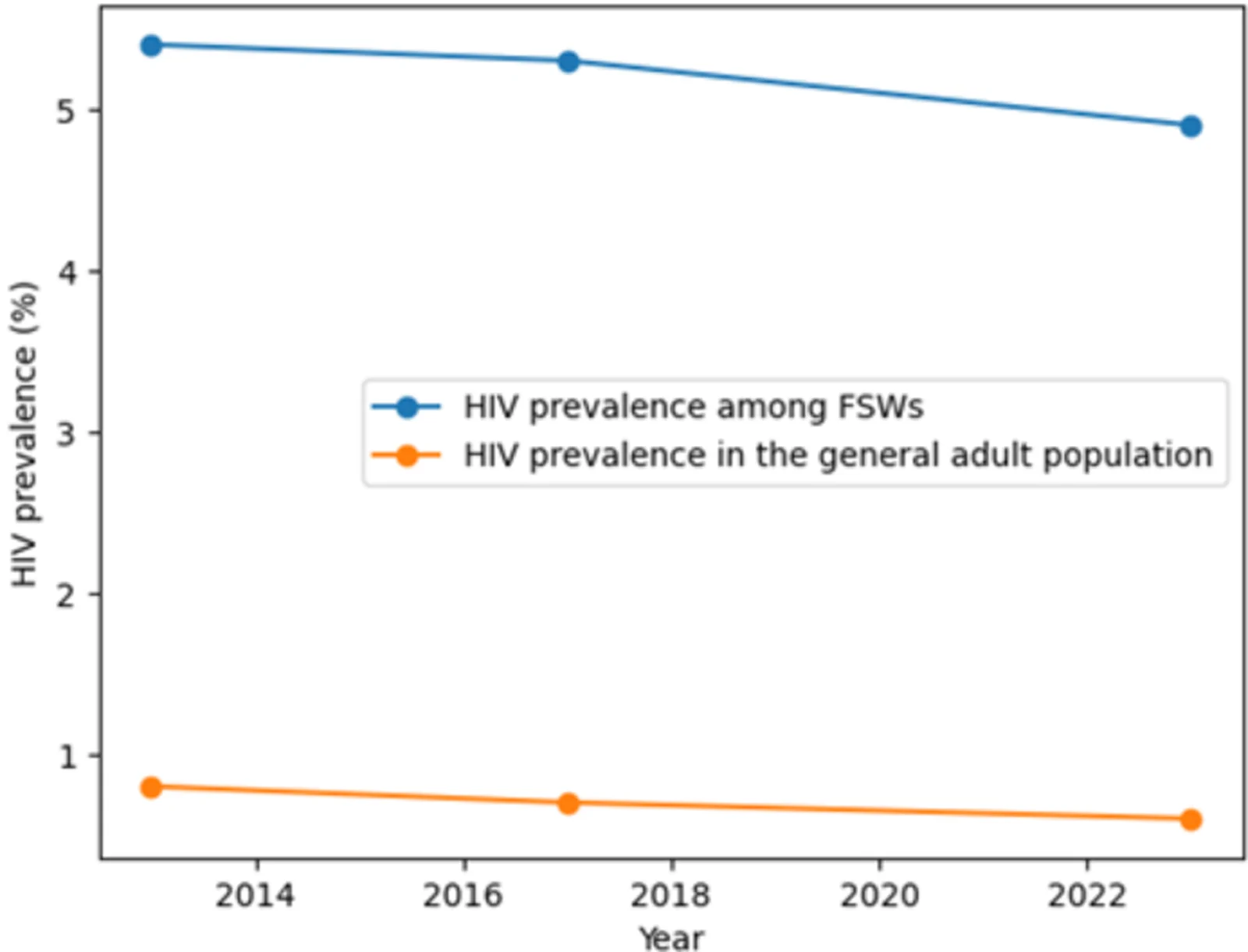
HIV prevalence among female sex workers in Cabo Verde, 2013-2023

**HIV prevalence trends among female sex workers:** across the three integrated bio-behavioural survey (IBBS) rounds the National HIV prevalence among FSWs was 5.4% in 2013, 5.3% in 2017, and 4.9% in 2023. Compared with general adult population prevalence estimates of approximately 0.8%, 0.7%, and 0.6%, respectively (Figure 1) [8]. Although this downward trend was not statistically significant (Cochran-Armitage test for trend, p = 0.21), it suggests gradual improvements in HIV prevention and service uptake over the decade. Notably, the most recent prevalence estimate remains nearly five times higher than the national adult HIV prevalence of less than 1%, underscoring the continued concentration of HIV within this key population.

**Figure 2:**
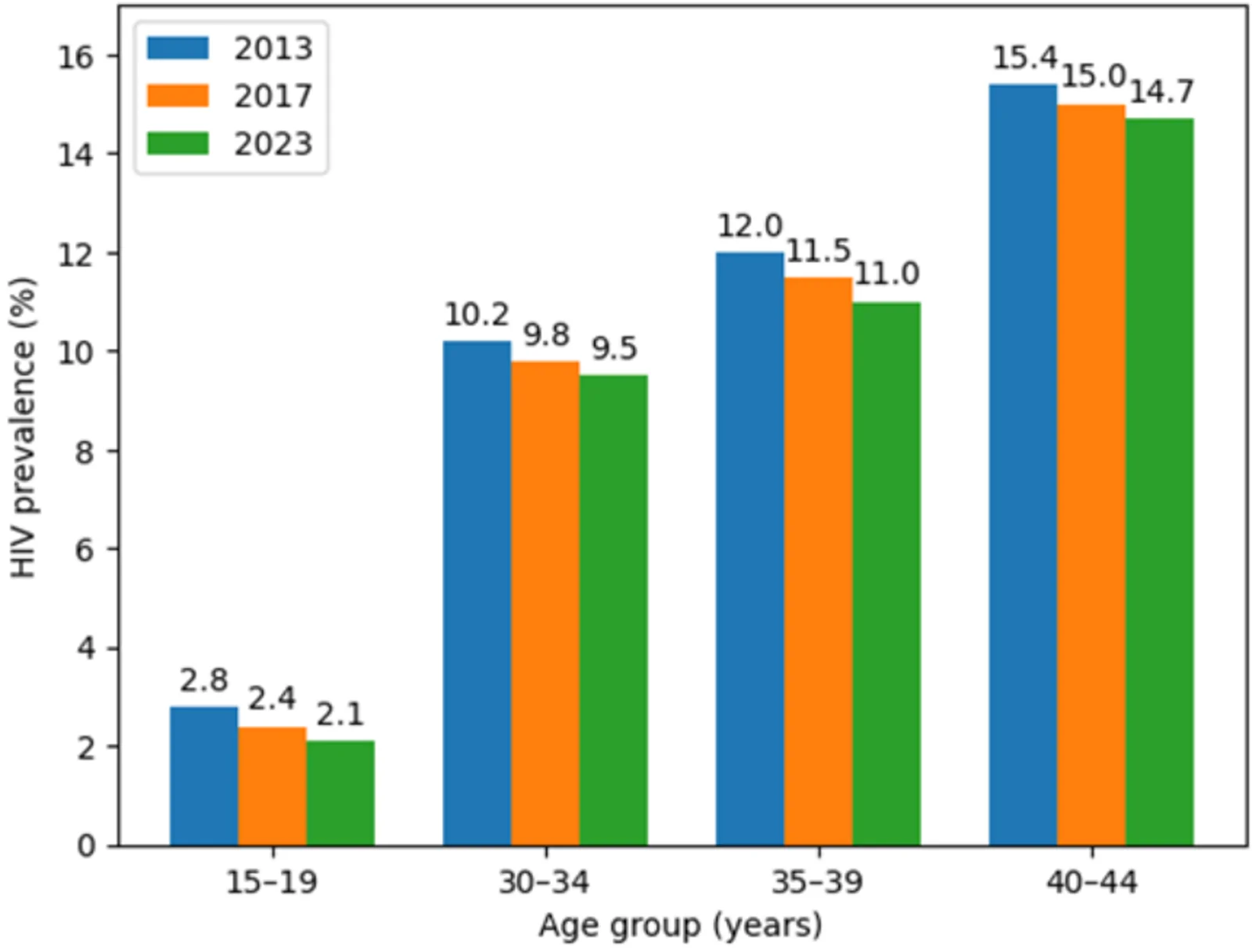
HIV prevalence with advancing age among female sex workers Cabo Verde, 2023

**Age-specific patterns of HIV prevalence:** HIV prevalence increased with age, reflecting cumulative exposure to HIV risk over time. In 2023, prevalence ranged from 2.1% among FSWs aged 15-19 years to 14.7% among those aged 40-44 years (Figure 2). Intermediate prevalence levels were observed among women aged 30-34 years (9.5%) and 35-39 years (11.0%). Bivariate analysis demonstrated a positive correlation between age and HIV prevalence (Spearman´s ρ = 0.22; p < 0.01). In multivariable logistic regression analysis, older age remained independently associated with HIV infection, with women aged 40-44 years having significantly higher odds of HIV infection compared with younger age groups (adjusted odds ratio [aOR] = 3.7; 95% confidence interval [CI]: 1.9-6.9). Older age was associated with higher HIV infection prevalence. These findings indicate increasing vulnerability with advancing age among FSWs.

**Geographic variation in HIV prevalence and condom use:** substantial geographic variation in both condom use, and HIV prevalence was observed across municipalities in 2023 (Table 2). Condom use with the last paying client was highest in Sal (95.7%) and lowest in Santa Catarina (66.7%). Correspondingly, HIV prevalence was lowest in Sal (1.2%) and highest in Santa Catarina (5.4%). Correlation analysis confirmed a statistically significant inverse relationship between municipality-level condom use and HIV prevalence (Spearman´s ρ = -0.18; p = 0.04), indicating that areas with lower reported condom use experienced higher HIV prevalence.

**Table 2:** trends in sexually risky behaviours among female sex workers in Cabo Verde, 2013-2023

Municipality	Condom use with last paying client (%)	HIV prevalence (%)
Sal	95.7	1.2
Praia	91.4	1.8
São Vicente	89.6	2.0
Boa Vista	88.3	2.4
Santa Cruz	83.5	3.1
São Filipe	80.2	3.6
Santa Catarina	66.7	5.4
Mean (SD)	85.0 (10.1)	2.8 (1.4)
Correlation (ρ)	–0.18 (p = 0.04)	—

**Figure 3:**
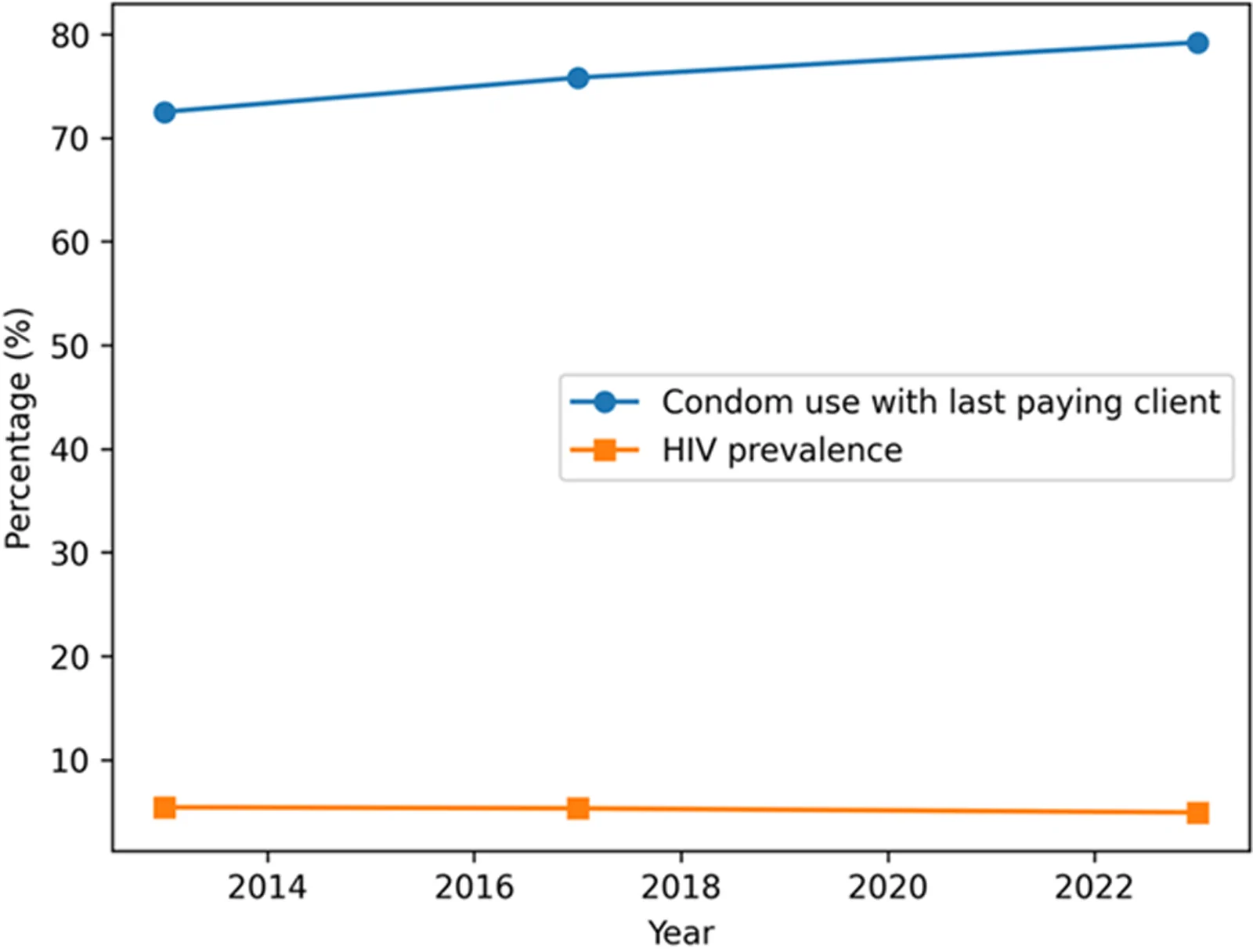
trends in condom use and HIV prevalence among female sex workers in Cabo Verde, 2013-2023

**Sexual risky behaviours and HIV infection:** reported condom use with the last paying client increased steadily over the three survey rounds, from 72.5% in 2013 to 75.8% in 2017 and 79.2% in 2023 (Table 3). Despite this overall improvement, marked variability persisted by municipality, with consistently lower condom use observed in Santa Catarina compared with tourism-driven municipalities such as Sal. In 2023, consistent condom use with all clients was reported by 68.4% of participants and was significantly associated with reduced odds of HIV infection. In multivariable analysis, FSWs reporting consistent condom use had a 40% lower likelihood of being HIV-positive compared with those reporting inconsistent use (aOR = 0.6; 95% CI: 0.3-0.9). Alcohol use was common among FSWs and showed a modest association with HIV prevalence. In 2023, HIV prevalence was 5.0% among women reporting alcohol use compared with 4.4% among non-users. Although this association was not statistically significant (Spearman´s ρ = 0.07; p = 0.14), alcohol consumption may indirectly increase HIV vulnerability through reduced condom negotiation and engagement in higher-risk sexual encounters. Temporal trends in condom use and HIV prevalence are illustrated in Figure 3, which demonstrates a steady increase in condom use alongside a gradual decline in HIV prevalence from 2013 to 2023.

**Table 3:** access to HIV testing services and association with stigma among female sex workers in Cabo Verde, 2013-2023

Variable	2013	2017	2023	Key findings
Condom use with last paying client (%)	72.5	75.8	79.2	Progressive increase across rounds; significant variability by municipality (highest in Sal: 95.7%; lowest in Santa Catarina: 66.7%)
Consistent condom use with all clients	—	—	68.4	Significantly associated with reduced HIV infection (aOR = 0.6; 95% CI 0.3—0.9)
HIV prevalence among alcohol users (%)	—	—	5.0	Alcohol use prevalent; modest correlation with HIV (ρ = 0.07; p = 0.14)
HIV prevalence among non-alcohol users (%)	—	—	4.4	Lower prevalence than alcohol users; not statistically significant

**Figure 4:**
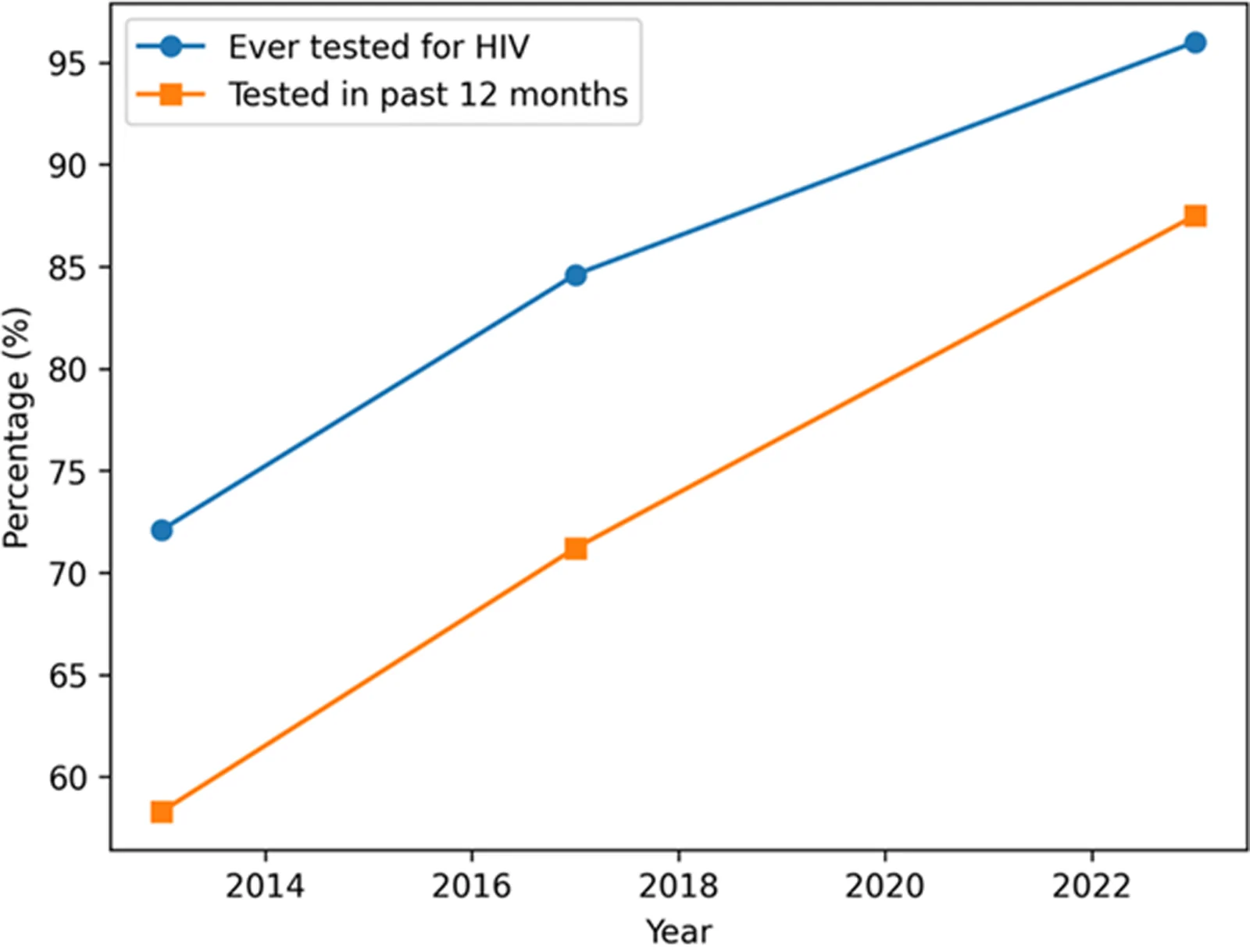
HIV testing coverage among female sex workers in Cabo Verde, 2013-2023

**Access to HIV testing and services:** access to HIV testing improved substantially over the study period (Table 4; Figure 4). The proportion of FSWs who reported ever having tested for HIV increased from 72.1% in 2013 to 84.6% in 2017 and reached 96.0% in 2023. Similarly, recent HIV testing (within the previous 12 months) rose from 58.3% in 2013 to 71.2% in 2017 and 87.5% in 2023. These findings indicate near-universal testing coverage by 2023 and reflect expanded outreach and integration of HIV testing services within community-based programs.

**Table 4:** correlation between HIV prevalence and selected sociodemographic and structural factors among female sex workers in Cabo Verde, 2023

Indicator	2013	2017	2023
Ever tested for HIV (%)	72.1	84.6	96.0
Tested in past 12 months (%)	58.3	71.2	87.5
Reported stigma or discrimination (%)	—	—	38.0
HIV prevalence among women reporting stigma (%)	—	—	6.8
Adjusted odds ratio for HIV among women reporting stigma (95% CI)	—	—	1.8 (1.1-3.0)

Despite high testing coverage, stigma and discrimination remained prevalent. In 2023, 38.0% of participants reported experiencing stigma, including family rejection, social exclusion, or discriminatory treatment in healthcare settings. HIV prevalence was higher among women reporting stigma (6.8%) compared with those who did not report such experiences. In multivariable analysis, experiencing stigma was independently associated with HIV infection, with nearly twice the odds of HIV positivity among affected women (aOR = 1.8; 95% CI: 1.1-3.0) (Table 4).

**Correlates of HIV prevalence: municipality, age, and education:** correlation analyses identified consistent associations between HIV prevalence and key sociodemographic and structural factors (Table 5). Municipality-level condom use was inversely correlated with HIV prevalence (Spearman´s ρ = -0.18; p = 0.04), confirming persistent geographic disparities. Age showed a strong positive correlation with HIV prevalence (Spearman´s ρ = 0.22; p < 0.01), consistent with the age-specific patterns described above. Educational attainment was strongly and inversely associated with HIV prevalence. In 2023, HIV prevalence was highest among women with no formal education (23.1%) and lowest among those with higher education (2.2%). This relationship was statistically significant (Spearman´s ρ = -0.27; p < 0.001) (Table 4). Lower educational attainment was therefore associated with substantially increased HIV vulnerability.

**Table 5:** the correlation of HIV prevalence with municipality, age, and education, with the Spearman's and p-value results, Cabo Verde, 2013-2023

	Variable	Spearman’s p	P-value
1	Municipality-level condom use vs HIV prevalence (2023)	-0.18	0.04
2	Age vs HIV prevalence (2023)	0.22	<0.01
3	Educational attainment vs HIV prevalence (2023)	-0.27	<0.001

**Multivariable regression analysis:** Table 6 presents the multivariable logistic regression analysis of factors associated with HIV infection among female sex workers across the three survey rounds. In pooled analyses, older age was the strongest predictor of HIV infection. Compared with women aged 15-24 years, those aged 35-44 years had more than three times higher odds of HIV infection (aOR = 3.5; 95% CI: 2.1-5.8). Educational attainment showed a strong inverse association with HIV infection. Women with no formal education had fivefold higher odds of being HIV-positive compared with those with secondary or higher education (pooled aOR = 5.0; 95% CI: 3.0-8.4). Inconsistent condom use was independently associated with HIV infection (pooled aOR = 1.7; 95% CI: 1.2-2.5). Alcohol use showed a positive but non-significant association across all survey rounds. In 2023, experiencing stigma or discrimination was independently associated with higher HIV prevalence (aOR = 1.8; 95% CI: 1.1-3.0), and this association remained significant in pooled analyses (aOR = 1.9; 95% CI: 1.2-3.1). Geographic location was also a significant predictor, with female sex workers in high-prevalence municipalities having more than twice the odds of HIV infection compared with those in lower-prevalence areas (pooled aOR = 2.4; 95% CI: 1.6-3.7).

**Table 6:** multivariable logistic regression of factors associated with HIV infection among female sex workers in Cabo Verde, 2013-2023 (RDS-weighted)

Variable	Category	2013 aOR (95% CI)	2017 aOR (95% CI)	2023 aOR (95% CI)	Pooled aOR (95% CI)
Age group (years)	15–24 (ref)	1.0	1.0	1.0	1.0
	25–34	1.8 (0.9–3.4)	2.1 (1.1–3.9)	2.4 (1.3–4.5)	2.1 (1.3–3.4)
	35–44	3.2 (1.6–6.3)	3.5 (1.8–6.7)	3.7 (1.9–6.9)	3.5 (2.1–5.8)
Education	Secondary+ (ref)	1.0	1.0	1.0	1.0
	Primary	1.9 (1.0–3.5)	2.2 (1.2–4.1)	2.5 (1.4–4.6)	2.2 (1.4–3.6)
	No formal education	4.6 (2.1–10.1)	4.9 (2.3–10.4)	5.3 (2.6–11.0)	5.0 (3.0–8.4)
Consistent condom use	Yes (ref)	1.0	1.0	1.0	1.0
	No	1.7 (0.9–3.1)	1.6 (0.9–2.9)	1.8 (1.1–3.0)	1.7 (1.2–2.5)
Alcohol use	No (ref)	1.0	1.0	1.0	1.0
	Yes	1.3 (0.7–2.4)	1.4 (0.8–2.6)	1.5 (0.9–2.7)	1.4 (0.9–2.1)
Experienced stigma	No (ref)	–	–	1.0	1.0
	Yes	–	–	1.8 (1.1–3.0)	1.9 (1.2–3.1)
Municipality	Low-prevalence (ref)	1.0	1.0	1.0	1.0
	High-prevalence	2.1 (1.1–3.9)	2.3 (1.3–4.2)	2.6 (1.5–4.6)	2.4 (1.6–3.7)

Note: all models adjusted for survey year and RDS weights; Ref = reference category; – = variable not collected in that round. CI: confidence interval; Aor: adjusted odds ratio

Overall, these findings demonstrate that HIV risk among FSWs in Cabo Verde is shaped by a combination of individual behaviours and structural determinants. Older age, lower educational attainment, persistent stigma, and geographic disparities in condom use contribute to ongoing HIV vulnerability, highlighting the need for targeted, multi-level interventions.

## Discussion

This study provides a rare decade-long analysis of HIV epidemiology among female sex workers (FSWs) in Cabo Verde, a small island developing state (SIDS) with a concentrated HIV epidemic. Findings from three consecutive integrated bio-behavioural surveys demonstrate that HIV prevalence among FSWs has remained persistently elevated at approximately 5% between 2013 and 2023, despite modest declines and substantial improvements in HIV service coverage. This prevalence remains markedly higher than that of the general population, where HIV prevalence is below 1%, underscoring the continued concentration of HIV among key populations and the challenges of achieving equitable epidemic control in small-island contexts.

The use of respondent-driven sampling introduces specific methodological challenges that must be acknowledged. Although RDS improves access to hidden populations such as female sex workers, it assumes random recruitment within social networks and accurate reporting of network size, assumptions that are rarely fully met in practice. Recruitment homophily, differential network connectivity, and the exclusion of highly marginalized individuals may bias prevalence estimates. These limitations highlight the importance of interpreting RDS-derived estimates as approximations rather than precise population parameters.

The persistence of elevated HIV prevalence among FSWs in Cabo Verde aligns with evidence from across West Africa, where sex workers continue to bear a disproportionate burden of infection despite national-level declines in HIV incidence. Studies in Côte d´Ivoire have reported HIV prevalence ranging from 15% to 20% among FSWs, compared with less than 3% in the general population [24]. Similarly, a national survey in Ghana estimated HIV prevalence among FSWs at approximately 11%, with substantial regional variation reflecting uneven prevention coverage [25]. Even in Senegal, frequently cited as a regional success story for HIV control, prevalence among sex workers remains above 7%, particularly in urban areas with limited-service reach [26]. While Cabo Verde´s prevalence is lower than that observed in many continental West African settings, the persistent gap between FSWs and the general population confirms ongoing structural and social vulnerabilities.

A pronounced age gradient in HIV prevalence was observed, with prevalence peaking at nearly 15% among women aged 40-44 years. This pattern mirrors findings from Nigeria and Burkina Faso, where older sex workers consistently exhibit higher prevalence due to cumulative exposure, longer duration in sex work, and limited access to prevention and treatment services earlier in their lives [27,28]. These findings highlight the importance of life-course approaches to HIV prevention that address both current risk and historical gaps in service access. Educational attainment emerged as a strong protective factor, with HIV prevalence substantially higher among women with no formal education compared with those who had completed higher levels of schooling. This inverse relationship is consistent with evidence from The Gambia and Sierra Leone, where FSWs with secondary education or higher demonstrate greater condom use, improved health literacy, and higher uptake of HIV services [29,30]. Education likely operates through multiple pathways, including enhanced negotiation power, economic opportunities, and access to information, underscoring its role as a structural determinant of HIV vulnerability. These findings suggest that educational and economic empowerment interventions should be considered integral components of HIV prevention strategies for FSWs.

Geographic disparities in HIV risk and prevention were evident across municipalities in Cabo Verde. Lower condom use and higher HIV prevalence were observed in municipalities such as Santa Catarina, while tourism-driven areas such as Sal demonstrated higher condom use and lower prevalence. Similar intra-country heterogeneity has been documented in Guinea-Bissau and Liberia, where urban centers with strong non-governmental organization presence report better prevention outcomes compared with peripheral regions with limited outreach [31,32]. In archipelagic states like Cabo Verde, inter-island mobility, transportation barriers, and uneven distribution of services likely exacerbate these disparities, highlighting the need for geographically tailored interventions.

The substantial expansion of HIV testing coverage among FSWs, from 72% in 2013 to 96% in 2023, represents a significant public health achievement, particularly within a SIDS context characterized by constrained healthcare infrastructure. Comparable gains have been reported in Mauritius and the Seychelles, where targeted testing initiatives have achieved coverage exceeding 90% among key populations [33]. However, high testing coverage alone is insufficient to eliminate disparities. Nearly 40% of FSWs in Cabo Verde reported experiencing stigma or discrimination, and such experiences were independently associated with higher odds of HIV infection. These findings are consistent with evidence from Jamaica and the Dominican Republic, where stigma in healthcare settings and social exclusion remain major barriers to sustained engagement in HIV prevention and care [1,34]. Persistent stigma thus continues to function as both a human rights violation and a structural driver of HIV transmission.

From a programmatic perspective, findings suggest the need for differentiated prevention strategies targeting specific subgroups. Older female sex workers (≥35 years), women with no formal education, and those working in high-prevalence municipalities such as Santa Catarina should be prioritized for intensified outreach. Interventions should include decentralized PrEP delivery through municipal clinics, peer-led education for low-literacy populations, and stigma-reduction training for healthcare workers in high-burden areas. Evidence from Kenya and South Africa demonstrates that PrEP is highly effective in reducing HIV incidence among women at high risk when delivered with adherence support and community engagement [35,36]. In other SIDS, such as the Bahamas and Trinidad and Tobago, PrEP implementation has begun among key populations, although coverage remains uneven, illustrating both feasibility and challenges relevant to Cabo Verde [37].

In addition, harm reduction approaches addressing alcohol and substance use warrant expansion. Studies from Nigeria and Sierra Leone have linked alcohol consumption to impaired condom negotiation and increased HIV acquisition risk among sex workers [37]. Addressing these risks requires integrated interventions that combine behavioral counseling, psychosocial support, and structural approaches within sex worker health services.

Finally, stigma reduction must be central to national HIV strategies. Structural interventions-including legal literacy, sensitization of healthcare workers, and community empowerment-have demonstrated effectiveness in improving prevention outcomes. In Benin, combined legal advocacy and peer-led interventions increased condom use and HIV testing among FSWs [38]. In Mauritius, a rights-based national HIV response integrating civil society organizations led to reductions in stigma and improvements in treatment adherence among sex workers and people who use drugs [39]. Cabo Verde, which has achieved notable public health milestones such as malaria elimination, is well-positioned to embed similar rights-based approaches into its HIV response. Although HIV incidence is the most appropriate indicator of program effectiveness, it could not be directly estimated in this study due to the absence of longitudinal follow-up and recency assays. Nonetheless, the very low prevalence observed among adolescents and young FSWs (15-19 years) suggests that most new infections likely occurred in earlier cohorts, before the scale-up of current prevention services. This pattern is consistent with declining incidence among younger sex workers and supports the effectiveness of recent prevention interventions, including expanded condom distribution and HIV testing.

**Study limitations:** this study has several important limitations that should be considered when interpreting the findings. First, the use of respondent-driven sampling (RDS), while appropriate for reaching hidden populations, is subject to methodological constraints. RDS relies on non-random seed selection and peer recruitment, which may introduce recruitment bias and homophily, whereby participants preferentially recruit others with similar characteristics. Although equilibrium was achieved for key variables, some subgroups of female sex workers, particularly those who are socially isolated or newly engaged in sex work, may be underrepresented.

Second, the cross-sectional design of the surveys precludes causal inference. Observed associations between HIV infection and factors such as age, education, stigma, and condom use should be interpreted as correlational rather than causal relationships. Third, behavioural data were self-reported and may be affected by social desirability and recall bias, particularly for sensitive topics such as condom use, substance use, and experiences of stigma. Despite the use of trained interviewers and confidential interview settings, underreporting of risk behaviours cannot be excluded. Fourth, survivorship bias may influence age-specific prevalence patterns. Older female sex workers who remain in sex work and are available for recruitment may differ systematically from those who exited sex work or died earlier, potentially leading to overestimation of HIV prevalence in older age groups. Fifth, high mobility across islands and municipalities may affect trend comparability over time. Changes in migration patterns, tourism flows, and sex work venues between survey rounds could influence the composition of the recruited samples. Finally, although the same standardized IBBS protocol was used across all three rounds, minor contextual adaptations and differences in field implementation may limit perfect comparability of trends over the decade.

Nonetheless, the findings demonstrate that HIV among female sex workers in Cabo Verde remains concentrated and shaped by intersecting behavioural, educational, geographic, and structural determinants. Evidence from West Africa and other island states indicates that biomedical interventions alone are insufficient, underscoring the need for comprehensive, equity-driven approaches that address both individual risk and the broader social context.

## Conclusion

HIV prevalence among female sex workers in Cabo Verde has remained persistently high over the past decade, despite substantial improvements in HIV testing coverage and condom use. The study identified significant associations between HIV infection and older age, lower educational attainment, experiences of stigma, and geographic disparities. These findings underscore the continued concentration of HIV among key populations and highlight the importance of equity-driven, rights-based, and geographically targeted prevention strategies. Given the cross-sectional nature of the data, results should be interpreted as associations rather than causal relationships.

### What is known about this topic

- Female sex workers experience a disproportionately high burden of HIV globally and in sub-Saharan Africa, with prevalence several times higher than in the general population despite expanded access to HIV testing and treatment services;
- Structural factors such as stigma, limited educational attainment, gender-based vulnerability, and uneven access to prevention tools contribute substantially to persistent HIV risk among female sex workers.

### What this study adds

- This study provides rare decade-long trend data from three consecutive integrated bio-behavioural surveys, demonstrating that HIV prevalence among female sex workers in Cabo Verde has remained persistently elevated over ten years despite near-universal HIV testing coverage;
- It identifies age, educational attainment, stigma, and geographic disparities in condom use as key determinants of HIV vulnerability in a small island developing state context, highlighting the need for equity-driven, geographically tailored, and rights-based HIV prevention strategies.
